# The Heart in Life Insurance

**Published:** 1900-04

**Authors:** John N. Upshaw

**Affiliations:** Richmond, Virginia, Emeritus Professor of Practice, Medical College of Virginia; Medical Examiner Equitable Life of New York and Union Mutual of Maine.


ATLANTA

### Journal-Record of Medicine.

Successor to Atlanta Medical and Surgical Journal, Established 1855, and Southern Medical Record, Established 1870.

VOL. II. APRIL, 1900. No. 1.

EDITORS { BERNARD WOLFF, M.D., DUNBAR ROY, M.D. — M. B. HUTCHINS, M.D., BUSINESS MANAGER.

PUBLISHED MONTHLY. OFFICE 305 AND 306 FITTEN BUILDING.

### *ORIGINAL COMMUNICATIONS.*

### THE HEART IN LIFE INSURANCE.*

BY JOHN N. UPSHAW, M.D., RICHMOND, VIRGINIA,

Emeritus Professor of Practice, Medical College of Virginia; Medical Examiner Equitable Life of New York and Union Mutual of Maine.

To do our whole duty to both the company which has commissioned us, to the agent and applicant, is often a difficult problem to solve, lest in guarding the rights of the company we do an injustice to the agent, and worse still, a wrong to the applicant. Believing that there are many cases in which there are abnormal conditions of the heart, and yet in which that organ is capable of and does carry on a normal circulation, and consequently in which the applicant has a first-class chance of measuring up to the requirement of living out his expectancy, I am moved to discuss

*Read before the Tri-State Societies of the Carolinas and Virginia, in Charleston, February 20, 21, 22, 1900.

WI
AT459H

these conditions; at the same time I am persuaded that conditions are overlooked and policies issued upon the recommendation of the examiner which should be turned down, and so the company saved from loss. The views herein expressed are the result of a long experience as an examiner in life insurance.

Probably the one condition upon which policies are refused most promptly and hopelessly is *heart murmurs,* and in many cases rightly so. But it must be borne in mind that, in the clinical experience of many men, the subject of a heart murmur lives out his days, dying eventually from some other cause.

First I would speak of *functional murmurs:* Any applicant applying for insurance, showing a hemic murmur, undoubtedly out to be postponed until an improved condition of his blood causes the murmur to disappear, and all the conditions of a normal circulation to return. I say *postponed,* not *unqualifiedly rejected.*

Again, some reflex conditions may cause spasm in the columna carnea of one valve, a transient source of irritation of some kind, by which temporarily there is an imperfect closure of the valve, producing a murmur; *re-examination* at short intervals should be the course pursued here, to determine positively whether the murmur is transient or organic. This reflex irritation may be digestive, may be from excessive use of tobacco, or overwork, etc.

There may be an arrangement of the columnæ carnea, or one of them, by which, in its passage through the ventricle, a portion of blood passing under the chord through a narrow space, produces a bruit, heard at the first sound of the heart; the valves are intact and healthy, circulation normal, pulse steady, the subject can make any amount of physical exertion without any cardiac or pulmonary distress. Careful physical examination fails to develop *any hypertrophy, any misplacement to the right.* Gerhard says *a bruit under such conditions as this is insignificant,* and yet this applicant is refused insurance. A case illustrative has come under my observation: A young man of fine physique, a physician, applied for insurance, was examined and the murmur overlooked by the examiner. In answer to the question "if he knew anything connected with his health, physical condition, etc., which would make him an extra-hazardous risk for insurance," he replied that he had a "heart murmur." He had been examined several years before in New York by a distinguished man, who detected the murmur, but expressed the opinion that it would not shorten his life. The case was referred to me by the Medical Director. I made a careful examination, found a curious bruit, *soft at first*, and *ending at the conclusion of the systole tike the twang of a violin string.* There was positively *no hypertrophy*, no misplacement in any direction. The man's physique was perfect, and he had never experienced any discomfort referable to the heart. I rated him as *first-class*, and recommended that a policy be issued, arguing that there could be no valvular lesion for the reasons mentioned above, and the company promptly issued a policy on a desirable plan for a large amount.

Another condition in which I believe the rule should be modified, those cases, which in childhood have been the subjects of an acute endocarditis, recovering with valvular lesion, valves hardened or thickened, having lost in elasticity, but because of the youth of the subject and consequent recuperative powers, compensatory hypertrophy sooner or later develops, a perfectly normal circulation results, and a previously feeble child grows to manhood unconscious of any organic affection of his heart, able, oftentimes, to perform hard labor. I have a patient whom I have had under observation since his fourth year; he was the victim of recurring attacks of rheumatism, and the complicating endocarditis; frail and delicate until his fifteenth year, now about twenty-five, has an hypertrophied heart, well-marked bruit of mitral insufficiency. Compensation is absolutely perfect. He has had no rheumatism nor endocarditis for at least fifteen years; is nearly six feet in height, muscles as hard as iron, and by occupation a locomotive fireman, work requiring intense strain and active, untiring exertion in a constrained position, and yet absolutely no discomfort from heart. Can the man who has a heart normal in size, valves, and action, boast of more physical endurance? And yet such subjects are turned down, barred from life insurance, and deprived of this safe means of providing for wife and children.

Of course when subjects have reached middle age, or even adult life, and have compensatory hypertrophy established, following upon an endocarditis and consequent valve lesion, no question arises, because in cases such as this, there is greater danger that compensation will fail.

But there is another side to this question: We examine applicants like one recently examined by me: Of fine physique, but overweight, excessive abdominal measurement, with tendency to obesity, but active for a man of sixty, and not aware of any cardiac defect. Pulse intermitted four beats to the minute, auscultation revealed reduplication of heart sounds. The man may live out his expectancy of a little under fifteen years, but the chance is against it; this is the insurance test; and I unhesitatingly declined to recommend the risk. Nor is this all. I am satisfied that more influence should be attached to overweight in men of middle age and over, if they be obese, because it gives rise to suspicion of fatty heart; and while it may be simply a deposition of fat upon the heart walls, and so not as dangerous as when interstitial, yet the chances are too strong that interstitial fatty change will develop long before the man can reach his expectancy. Especially is this true in these subjects, because the tendency to rheumatism, or the development of lithemia or gout, with consequent endocarditis, or what is more insidious and more probable, atheromatous change in the blood-vessels, and molecular fatty change in the heart walls, promptly places the risk in the doubtful class, at best.

When a murmur exists, there is little risk that it be overlooked; indeed, murmur is confidently diagnosed sometimes when it does not exist, due to the fact that the sound of air in the part of the lung overlying the heart is heard, and the examiner fails to determine the fact by making the applicant hold his breath for a moment, fails to look for evidence of hypertrophy or misplacement.

But a heart in which the walls have undergone organic change, the ventricle dilated, the pulse in the individual, perchance, not too frequent, though it usually would be, and lacking in tension and force, escapes suspicions, and a case which should be rejected is passed, and recommended as "first-class."

I would also emphasize the fact that no risk is first-class when the sound of the valves *fails in clearness* and is *ruffled*, showing *rigidity* and *loss of elasticity*, beginning degeneration perhaps, or an undue *accentuation* of valvular sound, showing, perchance, "a rift within the lute," a something wrong somewhere; the starting point of increased action in the heart muscle, to eventuate in a compensatory hypertrophy rendered necessary by renal change in the early pathologic process of some form, usually interstitial, of chronic nephritis, with a pulse tension which should always arouse our suspicions, of renal trouble, remembering that in chronic interstitial nephritis, albumin is not so constant nor abundant as in the other forms, especially the parenchymatous.

Alcohol as a factor bearing upon the heart in life insurance is most important. Of course the daily taking of alcohol in quantity exceeding Ainstie's limit of itself bars the applicant, but in those subjects who have been addicted to the alcoholic habit, and have reformed, either by simply giving up a habit which they are convinced is harmful, or by means of the gold cure, it must not be forgotten that it may, and probably has left behind its baleful effects; and I make bold to affirm that, to say the least, the chance of subtle tissue change in the heart walls, the liver, kidneys, or stomach, are of such a nature as to forbid such a case being rated better than fair only. And to go a step farther, if the reform has come about through the *gold cure*, the risk is more undesirable still, and the higher standard companies recognize this fact and do not care to write policies for such risks. I recently rejected an applicant, a physician, who had nine years before been an alcoholic, taken the gold cure, and had been steadfast in his reformation. I found a too rapid pulse, persistently over ninety to a hundred, and feeble; heart action feeble; prematurely grey; nervous. I was satisfied he had fatty interstitial change of the heart walls, and promptly rejected him, confirming the opinion of another gentleman who had previously examined him and declined him; yet he was examined subsequently for another old line company, recommended, and a policy issued.

The irregularities of the heart, which come from reflexes, digestive, uterine, etc., are not of so great importance so far as the heart is concerned. It may necessitate a temporary postponement, but the important fact to determine here is the significance and importance of the trouble which has given rise to the reflexes.

I have been impressed by the influence of tobacco. I can in almost every case tell if the applicant has been smoking or chewing, though not so readily in the latter, by the increased number of beats of the pulse, running it up to, and sometimes over, a hundred; significant, too, is the lack of arterial tension. I cannot think, as a practical deduction from these practical facts, that an organ several times a day subjected to such excitation as the result of over stimulation of the vagus and subsequent exhaustion can be exempt from hurtful consequences.

I have observed that in excessive users of tobacco, the heart, when enfeebled and depressed, fails to respond to the influence of heart tonics as promptly as under other conditions, except possibly the persistent administration of strychnia. Tobacco heart is unfortunately a practical entity, and sudden death from heart-failure in such subjects is not so uncommon as to preclude the gravest consideration in determining the value of a risk, when the applicant is a tobacco habitué. I believe that its influence for evil is as wide-spread and potent, as insidious and dangerous, as other narcotic poisons which are more condemned.

*210 West Grace St.*

